# ERAS Protocol Elements and Neurosurgery-Specific Considerations: A Narrative Review

**DOI:** 10.4274/TJAR.2026.2606

**Published:** 2026-08-28

**Authors:** Rudin Domi, Sarah Saxena, Joana Berger-Estilita, Daniele Guerino Biasucci, Başak Ceyda Meco, Krenar Lilaj, Gentian Huti, Idit Matot, Francesca Rubulotta, Anne Marie Camilleri Podesta

**Affiliations:** 1 Medical University of Tirana Faculty of Medicine, Department of Surgery, Anaesthesia and Intensive Care Unit, Tirana, Albania; 2 Harvard Medical School, Department of Anaesthesiology, Helora, Belgium; 3 University of Mons, Research Institute for Health Sciences and Technology, Department of Surgery, Mons, Belgium; 4 Salem-Spital, Hirslanden Hospital Group, Department of Anaesthesiology and Intensive Care, Bern, Switzerland; 5 University of Bern, Institute for Medical Education, Bern, Switzerland; 6 University of “Tor Vergata” Rome, Department of Clinical Science and Translational Medicine, Rome, Italy; 7 Ankara University Faculty of Medicine, Department of Anaesthesia and Intensive Care, Ankara, Türkiye; 8 American Hospital 3, Clinic of Anaesthesia and Intensive Care, Tirana, Albania; 9 Tel Aviv University Faculty of Medicine, Tel Aviv Medical Center, Division of Anaesthesiology, Intensive Care and Pain, Tel Aviv, Israel; 10 CHIRMED University Hospital, Department of Anaesthesia and Intensive Care, Catania, Italy; 11 Chair International Women in Intensive and Critical Care Medicine Network, iWIN, Catania, Italy; 12 Mater Dei Hospital, Clinic of Anaesthesia and Intensive Care, Msida, Malta

**Keywords:** Craniosynostosis, craniotomy, enhanced recovery after surgery, geriatric, neurosurgery, pituitary surgery, spine surgery

## Abstract

Enhanced recovery after surgery (ERAS) is a structured, evidence-based, multimodal perioperative framework designed to reduce surgical stress and accelerate recovery. Although these pathways are widely established across surgical specialties, their implementation in neurosurgery presents unique challenges because recovery-enhancing interventions must be balanced against neurological protection, haemodynamic stability, intracranial physiology, and procedure-specific safety considerations. This narrative review synthesises current evidence regarding ERAS implementation across cranial and spinal neurosurgical procedures, including elective craniotomy, spine surgery, pituitary surgery, vascular neurosurgery, and selected paediatric and geriatric populations. While available studies consistently demonstrate reductions in hospital length of stay, opioid consumption, and resource utilisation without increasing perioperative complications, interpretation of these findings is limited by substantial heterogeneity in protocol design, outcome definitions, and adherence reporting, and by the scarcity of long-term neurological and patient-reported outcomes. Beyond summarising existing evidence, this review proposes a conceptual framework distinguishing two fundamentally different ERAS paradigms within neurosurgery: a function-driven model predominantly applicable to spine surgery, where recovery acceleration is the principal objective, and a safety-constrained model characteristic of cranial neurosurgery, where neurological monitoring, intracranial pressure management, and haemorrhagic risk frequently limit implementation of conventional ERAS elements. This distinction provides a physiological and clinical rationale for procedure-specific adaptation rather than uniform application of these principles. The review further argues that the benefits of ERAS arise primarily from pathway standardisation, multidisciplinary coordination, and high protocol adherence rather than from isolated perioperative interventions. By identifying the limitations of current evidence and highlighting the need for neurosurgery-specific outcome measures, standardised reporting, and protocol stratification by surgical pathology, this review offers a framework for future research and more tailored implementation of ERAS pathways within neurosurgical practice.

**Main Points**

• Enhanced recovery after surgery (ERAS) in neurosurgery should be viewed as a multidisciplinary model of care in which standardised perioperative pathways and high adherence to protocols are the main drivers of improved outcomes.

• Current evidence shows that ERAS is both safe and feasible for craniotomy and spinal surgery without increasing perioperative complications.

• Consistent benefits include reduced length of hospital stay, decreased opioid consumption, earlier mobilization, and lower healthcare costs.

• ERAS protocols must be tailored to the specific procedure and patient population, as neurosurgical contexts vary significantly in their physiological and clinical priorities.

• Despite these advantages, the existing evidence base remains limited by substantial heterogeneity and a predominance of single-center studies, highlighting the need for multicenter research and evaluation of long-term outcomes.

## Introduction

Enhanced recovery after surgery (ERAS) comprises a structured, evidence-based, multimodal approach to perioperative care that integrates preoperative, intraoperative, and postoperative interventions to reduce surgical stress, improve patient safety, and accelerate recovery. Since its introduction in colorectal surgery, ERAS pathways have been successfully adopted across numerous surgical specialties, consistently reducing postoperative morbidity, hospital length of stay (LOS), healthcare costs, and opioid consumption while improving patient-centred outcomes.^1, 2^

Despite these advances, implementation of ERAS in neurosurgery has progressed more cautiously than in other surgical disciplines and remains less standardized. Spine surgery was the earliest neurosurgical domain to adopt ERAS principles, largely because its perioperative objectives—including pain control, early mobilisation, and functional recovery—align closely with conventional ERAS goals.^3, 4, 5^ In contrast, cranial neurosurgery involves additional physiological and clinical constraints, including preservation of neurological function, maintenance of intracranial homeostasis, precise haemodynamic management, prevention of seizures, and mitigation of haemorrhagic risk.^6, 7^ These considerations frequently necessitate modification or selective application of standard ERAS elements.

Although an expanding body of evidence supports the feasibility and safety of ERAS pathways across a wide range of neurosurgical procedures, including elective craniotomy, spine surgery, pituitary surgery, vascular neurosurgery, and in selected paediatric and geriatric populations, the current literature remains fragmented.^8, 9, 10, 11, 12^ Most studies focus on short-term metrics such as LOS, opioid consumption, and complication rates, while relatively little attention has been paid to long-term neurological recovery, patient-reported outcomes, protocol adherence, and the mechanisms through which ERAS interventions influence neurosurgical outcomes. Furthermore, existing reviews frequently treat neurosurgical ERAS as a homogeneous concept, despite substantial differences in physiological priorities, perioperative risks, and recovery objectives across neurosurgical subspecialties.

Rather than simply summarizing the literature, this narrative review proposes a conceptual framework that distinguishes two complementary but fundamentally different ERAS paradigms within neurosurgery. We suggest that spine ERAS is primarily a function-driven model, in which the principal goal is accelerated recovery and restoration of mobility, whereas cranial ERAS represents a safety-constrained model, in which recovery-enhancing interventions must be balanced against neurological monitoring requirements and procedure-specific risks. By examining ERAS through this lens, we aim to provide a more nuanced interpretation of current evidence, identify limitations of existing research, and highlight priorities for future protocol development and outcome assessment.

Accordingly, ERAS in neurosurgery should be viewed not as a uniform protocol but as a flexible, procedure-specific framework whose effectiveness depends on adapting core recovery principles to distinct neurosurgical physiological and clinical contexts.

## Methods of the Search Strategy

This narrative review was conducted using a structured literature search in PubMed, Scopus, and Google Scholar to identify studies published between January 2010 and January 2025. The search strategy combined medical subject headings and free-text keywords related to enhanced recovery pathways in neurosurgical practice. Search terms included “enhanced recovery after surgery,” “ERAS,” “perioperative medicine,” “neurosurgery,” “craniotomy,” “spine surgery,” “pituitary surgery,” “vascular neurosurgery,” “paediatric neurosurgery,” “craniosynostosis,” “geriatric neurosurgery,” “multimodal analgesia,” and “tranexamic acid(TXA).” Terms were combined using Boolean operators (and/or) and adapted to the indexing structure of each database. Representative search strings included (“enhanced recovery after surgery” or “ERAS”) and (“neurosurgery” or “craniotomy” or “spine surgery”), with additional procedure-specific searches performed for specialised populations and interventions.

The review sought to address three principal clinical questions: (1) the effectiveness of ERAS pathways across different neurosurgical subspecialties; (2) the extent to which ERAS protocols require adaptation according to procedure-specific physiological and safety considerations; and (3) the current evidence gaps limiting broader implementation and standardisation of neurosurgical ERAS programmes.

Eligible publications addressing ERAS concepts, protocols, implementation strategies, or outcomes in neurosurgical populations included randomised controlled trials, prospective and retrospective observational studies, systematic reviews, meta-analyses, clinical practice guidelines, and high-quality narrative reviews. Studies were limited to human subjects and to publications in English. Editorials, commentaries, conference abstracts, letters without original data, duplicate publications, and studies not directly related to neurosurgical ERAS pathways were excluded.

Titles and abstracts were independently screened by three authors, and full-text assessment of potentially eligible articles was then performed. Disagreements regarding study eligibility were resolved through discussion and consensus. Attention was paid to studies reporting clinically relevant outcomes, including length of hospital stay, postoperative complications, opioid consumption, mobilisation, readmission rates, patient satisfaction, functional recovery, and neurological outcomes.

Because of substantial heterogeneity in study design, patient populations, protocol composition, outcome definitions, and reporting methods, quantitative pooling of data was not considered appropriate. Consequently, findings were synthesised using a narrative approach, with an emphasis on identifying recurring themes, procedure-specific adaptations, areas of consensus, and unresolved controversies.

**Figure 1:**
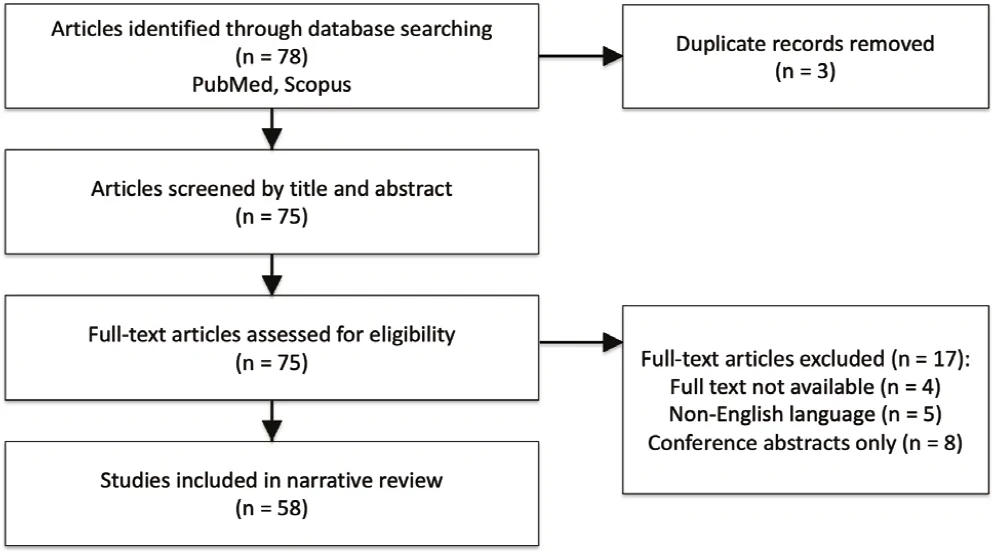
Study selection flow diagram.

As this work was designed as a narrative rather than a systematic review, no formal risk-of-bias assessment or evidence grading process was performed. Nevertheless, methodological quality was considered during evidence interpretation by prioritising higher-level evidence, including randomised trials, systematic reviews, meta-analyses, and clinical guidelines. The strength of conclusions was therefore based on the consistency of findings across studies, methodological rigor, and relevance to neurosurgical practice. A total of 58 studies met the eligibility criteria and were included in the final synthesis (Figure 1).

A limitation of this review is the absence of systematic application of formal quality-assessment and risk-of-bias tools, reflecting its narrative methodology. Although higher-level evidence was preferentially considered during synthesis, the conclusions remain dependent on the quality and heterogeneity of the available literature and should therefore be interpreted with appropriate caution.

## Results and Evidence Synthesis

### ERAS Protocols for Craniotomy: Evidence, Components, and Outcomes

The application of ERAS principles to craniotomy remains one of the most challenging areas in neurosurgical perioperative care. Unlike spinal surgery, cranial surgery poses unique constraints related to neurological protection, intracranial dynamics, haemodynamic stability, seizure risk and haemorrhagic complications. Consequently, ERAS protocols for craniotomy require substantial adaptation and cautious validation.

### Conceptual foundations and early frameworks

The first structured effort to adapt ERAS principles to craniotomy was undertaken by Hagan et al.,^13^ who applied the grading of recommendations assessment, development and evaluation methodology to evaluate both evidence quality and recommendation strength.The proposed framework incorporated 17 perioperative components, including patient counselling, nutritional optimisation, thromboprophylaxis, analgesia, normothermia, fluid management, early mobilisation, and audit processes. Although the framework represented an important step toward standardising perioperative care in cranial neurosurgery, its evidentiary foundation was uneven. Several recommendations were supported by moderate- to high-quality evidence, whereas others were largely derived from extrapolation of data from surgical populations outside neurosurgery. This finding highlighted a fundamental challenge in the early development of ERAS protocols for craniotomy: the need to balance physiological plausibility and established perioperative principles against the relative scarcity of procedure-specific evidence.

Table 1 summarises the levels of evidence and strengths of recommendation for individual ERAS components, as proposed by Hagan et al.^13^

Importantly, this framework established the conceptual rationale for ERAS implementation in cranial surgery, but also identified critical gaps that require prospective validation before widespread adoption could be justified.

### Feasibility and safety of ERAS in elective craniotomy

Subsequent investigations have shifted from conceptual development toward evaluating whether ERAS pathways can be implemented safely and effectively in clinical practice. Early evidence from Elayat et al.^14^ demonstrated a lower proportion of patients requiring prolonged intensive care unit (ICU) admission (>48 h) following ERAS implementation, although no significant reduction in overall hospital LOS was observed. While interpretation is constrained by the study’s non-randomised design and limited sample size, the findings provide initial evidence that ERAS principles could be incorporated into elective craniotomy pathways without compromising patient safety.

Further studies have strengthened this observation. Feng et al.^15^ reported improved postoperative recovery parameters without an increase in complications among patients managed within an ERAS framework, while Kaewborisutsakul et al.^16^ demonstrated that greater compliance with ERAS components was associated with superior recovery outcomes and shorter hospitalisation. The latter finding is particularly important because it suggests that implementation fidelity may be as influential as protocol composition itself. Collectively, these studies indicate that ERAS pathways can be safely implemented across diverse elective craniotomy populations, with no consistent evidence of increased perioperative morbidity. The consistency of safety findings across different institutions and study designs represents one of the most robust observations in the existing literature.

### Effects on LOS, mobilisation, and resource utilisation

Hospital LOS remains the most consistently reported outcome in studies evaluating ERAS after craniotomy. In a controlled implementation study involving glioma patients, Wang et al.^17^ reported shorter LOS and earlier mobilisation among patients managed within an ERAS pathway. Because patient allocation was based on treatment period rather than randomisation, temporal and practice-related confounding cannot be excluded. Similar reductions in LOS were subsequently reported in both retrospective and randomised studies.^15, 18^

Although the magnitude of LOS reduction varies across studies, the direction of effect has been remarkably consistent. This consistency suggests that accelerated recovery and earlier discharge represent genuine benefits of ERAS implementation rather than isolated institutional findings. Notably, studies reporting the most favourable outcomes generally incorporated early mobilisation, structured discharge planning, multimodal analgesia, and minimisation of unnecessary ICU utilisation, suggesting that coordinated pathway integration rather than any single intervention may drive improvements in perioperative efficiency.

In addition to reducing LOS, ERAS pathways have been associated with lower ICU utilisation and earlier mobilisation.^14, 15, 16, 17, 18^ However, conclusions regarding economic benefit should be interpreted cautiously. While shorter hospitalisation implies potential cost savings, formal economic analyses remain limited, and cost-related outcomes have not been systematically evaluated across studies.

### Pain control, opioid use, and patient-reported outcomes

Optimisation of postoperative pain control is one of the most clinically relevant components of craniotomy ERAS pathways. Evidence from randomised studies indicates that ERAS protocols can improve multiple patient-centred recovery outcomes beyond traditional surgical metrics.

In a randomised controlled trial involving 151 patients, Wang et al.^18^ demonstrated reductions in pain intensity, postoperative nausea and vomiting (PONV), opioid consumption, and hospitalisation costs among patients managed within an ERAS programme. Similarly, Liu et al.^19^ reported improved patient satisfaction and enhanced recovery measures without an increase in complications, while Qu et al.^20^ observed lower postoperative pain scores and shorter LOS in ERAS-treated patients.

A notable finding across these studies is the consistent improvement in pain-related outcomes despite variations in protocol composition. This suggests that the benefit is unlikely to be attributable to a specific analgesic intervention. Rather, multimodal opioid-sparing strategies, including scalp nerve blocks, non-opioid analgesics, and structured PONV prophylaxis, appear to exert complementary effects that facilitate recovery. These observations support the concept that ERAS functions as an integrated care model in which cumulative gains from multiple interventions may be more important than the effect of any individual component.

The adaptability of ERAS principles is further illustrated by the work of Chen et al.,^21^ who successfully applied an ERAS-oriented strategy for awake craniotomy, using monitored anaesthesia care and scalp blocks, demonstrating that protocolised recovery pathways can be incorporated even into highly specialised cranial procedures.

### Protocol composition and heterogeneity

Despite broadly favourable outcomes, substantial heterogeneity exists among published craniotomy ERAS protocols. Variations in carbohydrate loading practices, fluid management strategies, thromboprophylaxis timing, analgesic approaches, mobilisation targets, and nutritional interventions complicate direct comparisons between studies and limit the ability to identify the specific components responsible for the observed benefits.

This limitation was highlighted in the systematic review by Zangi et al.,^22^ which confirmed improvements in LOS and recovery-related outcomes but also emphasised considerable methodological heterogeneity, reliance on predominantly single-centre studies, and a lack of protocol standardisation. Although the overall direction of evidence favours ERAS implementation, the relative contribution of individual pathway elements remains insufficiently defined.

Nevertheless, some patterns emerge across the literature. Early mobilisation, multimodal opioid-sparing analgesia, structured PONV prophylaxis, standardised perioperative care pathways, and avoidance of unnecessary ICU admission are among the components most consistently associated with improved recovery outcomes. In contrast, evidence supporting the independent contribution of interventions such as preoperative carbohydrate loading, specific fluid management strategies, thromboprophylaxis timing, and nutritional optimisation remains limited and is frequently extrapolated from broader surgical populations. As a result, current evidence is stronger for the ERAS pathway as a comprehensive perioperative strategy than for many of its individual components.

### Summary of evidence and evidence mapping

Current data consistently support the feasibility and safety of ERAS pathways in elective craniotomy and suggest meaningful improvements in short-term recovery outcomes, particularly with respect to mobilisation, pain control, ICU utilisation, and hospital LOS.

Several conclusions can therefore be considered relatively well established. ERAS protocols can be implemented safely without increasing perioperative complications, and can facilitate earlier mobilisation, improve patient-reported recovery metrics, reduce opioid requirements, and shorten hospitalisation in selected elective craniotomy populations. These findings have been reproduced across observational studies, randomised trials, and systematic reviews.^14, 15, 16, 17, 18, 19, 20, 21, 22^

However, important uncertainties remain. It remains unclear which individual ERAS components contribute most to the observed benefits, whether outcomes are driven primarily by specific interventions or overall protocol adherence, and how findings can be generalised across different neurosurgical populations and healthcare systems. Furthermore, most available studies focus on short-term perioperative outcomes, leaving the effects on long-term neurological recovery, quality of life, functional independence, and cost-effectiveness insufficiently defined.

Future research should therefore prioritise multicentre validation studies, greater protocol standardisation, and evaluation of patient-centred long-term outcomes. Such work will be essential to determine not only whether ERAS improves recovery after craniotomy, but also which components provide the greatest value and should be prioritised in routine clinical practice. Table 2 summarises the study characteristics and ERAS components.

### ERAS Protocols in Specific Neurosurgical Situations

Neurosurgery encompasses heterogeneous patient populations and procedures that differ substantially from the surgical settings in which ERAS protocols were originally developed. Consequently, successful implementation often requires adaptation of core ERAS principles rather than strict adherence to a uniform protocol. Across paediatric, geriatric, pituitary, and vascular neurosurgery, the literature suggests that, while the overarching goals of reducing physiological stress and facilitating recovery remain consistent, the interventions and outcome priorities differ based on patient characteristics and procedural risks.^23^ These specialised applications highlight both the flexibility of ERAS principles and the need for procedure-specific strategies.

### Paediatric neurosurgery

Paediatric neurosurgery presents unique challenges for ERAS implementation due to age-dependent physiology, limited physiological reserve, developmental considerations, and the central role of caregivers in perioperative care. Consequently, paediatric ERAS pathways prioritise physiological stability and family-centred care rather than accelerated discharge.^24^

The most structured experience pertains to craniosynostosis surgery. A protocol incorporating standardised haemodynamic management, normothermia maintenance, multimodal analgesia, and blood-conservation strategies was associated with reduced opioid consumption, shorter hospitalisation, and fewer postoperative complications, although statistical significance was limited by sample size.^25^

Available evidence suggests that temperature regulation, blood loss prevention, caregiver involvement, and structured pain assessment are among the most relevant ERAS components in this population. In contrast, traditional adult ERAS targets such as rapid discharge appear to be less important. However, evidence remains limited, and the relative contribution of individual interventions is uncertain.^24, 25^

### Geriatric neurosurgery

The growing number of elderly patients undergoing neurosurgical procedures has increased interest in ERAS strategies tailored to frailty, multimorbidity, and reduced physiological reserve.^26^ Given the strong influence of baseline functional status on recovery, current recommendations emphasise assessment of frailty, cognitive function, nutritional status, and social support.^26^ Several ERAS elements may require modification in this population; for example, carbohydrate loading may be limited by impaired glucose tolerance, while pharmacological thromboprophylaxis must be balanced against haemorrhagic risk.^26^ Observational studies suggest that protocols incorporating frailty assessment, minimisation of invasive devices, multimodal analgesia, delirium prevention, and early mobilisation may improve functional recovery and reduce LOS without increasing complications.^27^ Nevertheless, evidence remains largely observational, and uncertainty persists regarding which interventions contribute most substantially to improved outcomes.

### Pituitary surgery

Pituitary surgery represents one of the clearest examples of procedure-specific ERAS adaptation. In contrast to conventional ERAS pathways, recovery priorities are centred on endocrine stability, fluid-electrolyte balance, and neurological safety rather than on gastrointestinal recovery alone.^28^

Across published studies, several components appear to be consistently important, including preoperative endocrine optimisation, minimally invasive endoscopic approaches, goal-directed fluid therapy, avoidance of routine nasal packing and of lumbar drains, structured patient education, and intensive postoperative endocrine monitoring.^28, 29, 30^ These interventions address the unique physiological challenges of pituitary surgery and distinguish pituitary ERAS pathways from broader cranial ERAS models.

Barrette et al.^29^ identified 19 recommendations for transsphenoidal surgery; more than one-third were supported by low-quality evidence, highlighting the need for further research. Similarly, studies by Pan et al.,^30^ Liu et al.,^31^ and Hughes et al.^32^ demonstrated shorter hospitalisation, improved recovery, high patient satisfaction, and the feasibility of ambulatory management in selected patients. Although protocol composition varied, the consistency of findings suggests that multidisciplinary care, endocrine surveillance, and patient education are among the most impactful ERAS elements in this setting.

### Intracranial aneurysm surgery

ERAS pathways for unruptured intracranial aneurysm surgery require substantial modification because neurological protection takes precedence over accelerated recovery.^12^ Key adaptations include optimisation of blood pressure, careful antiplatelet management, selective use of carbohydrate loading and thromboprophylaxis, intraoperative neurophysiological monitoring, and strict postoperative haemodynamic and neurological surveillance.^12^ These measures reflect the need to balance ERAS principles with procedure-specific safety considerations.

Evidence remains more limited than that for elective craniotomy or pituitary surgery. National-level data suggest that ERAS principles can be incorporated safely without increasing neurological complications,^12^ while the randomised study by Pandit et al. demonstrated shorter ICU stay, lower intraoperative fentanyl requirements, and higher patient satisfaction despite no significant reduction in overall LOS.^33^ These findings suggest that outcomes such as ICU utilisation and patient experience may be more sensitive measures of ERAS success in neurovascular surgery than hospital LOS alone.

### Summary of evidence and evidence mapping

Across specialised neurosurgical populations, ERAS implementation appears feasible and safe when protocols are adapted to procedure-specific physiological priorities. However, the literature consistently demonstrates that a uniform approach is unlikely to be appropriate. Instead, successful implementation depends on targeting the dominant determinants of recovery within each population, including physiological stability in paediatric patients, prevention of frailty and delirium in geriatric patients, endocrine homeostasis in pituitary surgery, and neurological protection in aneurysm surgery.

Several conclusions are reasonably well established. Tailored ERAS pathways improve perioperative efficiency and recovery without increasing complications. Multidisciplinary care, patient education, early mobilisation when appropriate, and protocol adherence emerge as recurring determinants of success across populations.^23, 24, 25, 26, 27, 28, 29, 30, 31, 32, 33^

Nevertheless, important uncertainties remain. Evidence is predominantly derived from single-centre studies; protocol heterogeneity is substantial; comparative data identifying the most influential ERAS components are limited. Furthermore, long-term neurological, functional, and quality-of-life outcomes remain insufficiently studied, highlighting priorities for future research.

### ERAS in Spine Surgery: Elements, Evidence, and Outcomes

ERAS protocols were adopted earlier in spine surgery than in cranial neurosurgery, and consequently have accumulated a larger evidence base. Spine surgery, therefore, represents the most mature neurosurgical application of ERAS as a perioperative care model integrating optimisation, standardisation, and multidisciplinary coordination to enhance recovery. Nevertheless, substantial heterogeneity in protocol composition, surgical case-mix, adherence reporting, and outcome definitions limits direct comparison across studies and complicates identification of the most influential ERAS components.^34, 35^

A useful framework is to consider spine ERAS across the preoperative, intraoperative, and postoperative phases, while recognising that observed benefits likely arise from coordinated pathway implementation rather than from any single intervention.^34, 35^ The ERAS Society consensus for lumbar spinal fusion provides the most comprehensive evidence-based framework currently available.^36^

### Preoperative spine ERAS: risk modification and expectation management

Preoperative ERAS strategies focus on reducing modifiable risk factors and improving patient engagement. Core elements include patient education, optimisation of comorbidities, nutritional assessment, anaemia correction, and smoking and alcohol cessation.^36, 37^ Although high-quality studies evaluating individual components are limited, these interventions are consistently incorporated into successful ERAS pathways and considered important enablers of postoperative mobilisation and recovery.^36, 37^

### Intraoperative spine ERAS: homeostasis, blood conservation, and minimising “recovery barriers”

Intraoperative ERAS strategies aim to preserve physiological homeostasis, minimise surgical stress, and remove barriers to recovery. Common components include normothermia, goal-directed fluid therapy, appropriate antimicrobial prophylaxis, minimisation of drains and urinary catheters, and, when appropriate, minimally invasive surgical techniques.^36, 37^ Among these interventions, blood conservation strategies and the avoidance of unnecessary postoperative devices appear to be most consistently associated with improved recovery and earlier mobilisation.

### Postoperative spine ERAS: mobilisation, catheter/drain avoidance, and rehabilitation

Postoperative ERAS pathways prioritise early mobilisation, early oral intake, multimodal analgesia, PONV prevention, and prompt removal of drains and urinary catheters.^37^ These interventions act synergistically to facilitate functional recovery and discharge readiness.

Across observational studies, the most consistent findings are reductions in hospital LOS and opioid consumption.^38, 39, 40^ Similar benefits have been observed in elderly and complex-fusion populations, suggesting that the effectiveness of ERAS extends beyond low-risk procedures.^39^ However, the magnitude of LOS reduction varies according to baseline institutional practices and discharge pathways.^40^

The impact on postoperative complications is less consistent. While some studies report lower complication rates, others demonstrate comparable outcomes between ERAS and conventional care.^38, 40^ Importantly, available evidence consistently indicates that ERAS does not increase adverse events.^38, 39, 40^ In contrast, readmission rates and pain scores often remain unchanged despite reductions in opioid consumption and LOS, suggesting that ERAS primarily improves perioperative efficiency and functional recovery rather than all postoperative outcomes.^40^

Overall, current evidence suggests that early mobilisation, multimodal analgesia, minimisation of catheters and drains, and pathway adherence are among the ERAS components most consistently associated with improved outcomes in spine surgery (Table 3).

### TXA as a spine ERAS blood conservation strategy

Blood conservation is one of the most extensively studied intraoperative ERAS domains in spine surgery, with TXA being one of the interventions most strongly supported by evidence.

### Efficacy of TXA

Multiple meta-analyses consistently demonstrate that intravenous TXA reduces intraoperative blood loss and transfusion requirements across complex spine procedures.^41, 42, 43, 44, 45^ The consistency of findings across randomised and pooled analyses suggests that TXA is one of the few individual ERAS interventions with reproducible and clinically meaningful benefits.

### Dosing Uncertainty of TXA

Despite consistent efficacy, optimal dosing remains uncertain. Although higher-dose regimens may provide greater blood-loss reduction, substantial heterogeneity in surgical procedures, bleeding risk, and co-interventions limits definitive recommendations regarding the ideal dosing strategy.^46, 47, 48^

### TXA Safety

The principal concern surrounding TXA remains thromboembolic safety. Current evidence generally demonstrates no significant increase in thromboembolic events, although many studies may be underpowered to detect rare complications.^49^ Consequently, while TXA appears safe in most patients and is increasingly incorporated into ERAS pathways, a risk-stratified approach remains appropriate, particularly in patients with elevated thromboembolic risk.^49^

### Analgesia as the functional “engine” of spine ERAS

Multimodal analgesia is arguably the most important postoperative component of spine ERAS because effective pain control directly influences mobilisation, rehabilitation, and discharge readiness.^50, 51^ Evidence consistently supports opioid-sparing multimodal strategies, with network meta-analysis demonstrating superior reductions in opioid consumption and pain scores when multiple analgesic classes are combined.^52^ These findings reinforce a central ERAS principle: recovery benefits arise from synergistic multimodal approaches rather than reliance on any single drug. Intravenous lidocaine may reduce opioid requirements and improve longer-term recovery outcomes, although concerns regarding toxicity and limited monitoring data have restricted widespread adoption.^53, 54^ Meta-analytic evidence supports ketamine as an effective opioid-sparing adjunct in adult spine surgery, although benefits appear to be influenced by timing of administration and the patient population.^55, 56^ Both agents have mechanistic and clinical rationales as components of multimodal analgesia, but spine-specific ERAS evidence remains less robust than that for ketamine or TXA.^57, 58^

Collectively, the available evidence indicates that multimodal analgesia is a major driver of ERAS success in spine surgery. However, uncertainty remains regarding the optimal combination of agents and dosing strategies, as well as their relative contribution to recovery outcomes.

### Summary of evidence and evidence mapping

Current evidence supports the implementation of ERAS in spine surgery as safe and effective, particularly in reducing LOS, opioid consumption, and resource utilisation. Among individual interventions, multimodal analgesia, early mobilisation, avoidance of unnecessary drains and catheters, and TXA-supported blood conservation appear to have the strongest evidence base.^34, 35, 36, 37, 38, 39, 40, 41, 42, 43, 44, 45, 46, 47, 48, 49, 50, 51, 52, 53, 54, 55, 56, 57, 58^

However, important uncertainties remain. Protocol heterogeneity, variable reporting of adherence, and differences in outcome definitions limit the identification of the relative contributions of individual ERAS components. Furthermore, while short-term recovery benefits are well established, evidence regarding long-term functional outcomes, quality of life, and cost-effectiveness remains comparatively limited. Future studies should focus on standardising protocols and determining which ERAS interventions provide the greatest incremental benefit across different spine surgery populations.

## Discussion

This narrative synthesis demonstrates that ERAS pathways in neurosurgery are feasible, safe, and associated with consistent improvements in perioperative efficiency. The most frequently reported benefits include reduced hospital LOS, decreased opioid consumption, and improved coordination of perioperative care.^1, 2, 3, 4, 5, 6, 7,16,18,34,35,37,40^ However, LOS should not be interpreted as a direct measure of recovery, because it is influenced by organisational, social, and health-care system factors. More clinically meaningful outcomes include functional recovery, neurological preservation, avoidance of complications, quality of recovery, and patient satisfaction, although these remain less consistently reported across studies. These benefits are most evident in spine surgery, where ERAS implementation is more mature, but are increasingly observed in elective cranial procedures and in selected high-risk populations when neurosurgery-specific adaptations are applied.^13, 14, 15, 16, 17, 18, 19, 20, 21, 22,24, 25, 26, 27, 28, 29, 30, 31, 32^

### ERAS as a Care-Delivery Model

A key insight emerging from this review is that ERAS benefits arise primarily from changes in care delivery rather than from isolated interventions. Multimodal analgesia, standardised perioperative workflows, early mobilisation, and avoidance of unnecessary invasive devices act synergistically to reduce variability and facilitate recovery.^16, 34, 36, 37^ Evidence that improved outcomes are associated with higher ERAS adherence further supports interpreting ERAS as an integrated care model in which implementation quality is as important as protocol content.^16, 28, 40^

This framework is particularly relevant in neurosurgery, where uncoordinated application of individual interventions may carry risk. ERAS pathways mitigate these risks by embedding individual interventions within coordinated multidisciplinary perioperative planning that balances recovery objectives with neurological safety.^13, 36^ Importantly, the value of ERAS should be assessed not only by efficiency metrics but also by its ability to preserve neurological function, enhance patient experience, and support high-quality recovery.

### LOS

The reduction in LOS should be interpreted with caution. Discharge criteria vary substantially across institutions, and LOS is influenced by non-medical factors such as social support, rehabilitation availability, and healthcare infrastructure. The inability to blind care providers and discharge planners may further amplify apparent LOS benefits. These limitations suggest that LOS should be interpreted primarily as a process outcome reflecting care coordination and system efficiency, rather than a direct surrogate for improved neurological recovery or long-term functional outcomes. Future studies should therefore place greater emphasis on patient-centred endpoints, including functional status, quality of recovery, neurocognitive outcomes, and patient-reported measures.

### Cranial versus Spine ERAS

Fundamental differences between cranial and spinal surgery preclude a uniform ERAS standardisation. Spine ERAS pathways are largely function-driven, with outcomes closely linked to pain control, early mobilisation, blood conservation, and rehabilitation engagement.^34, 35, 36, 37, 38, 39, 40^ In this context, opioid-sparing multimodal analgesia and blood loss mitigation strategies, particularly TXA, play a central role in accelerating recovery.^43,45, 46, 47,52^

Collectively, the spine surgery literature suggests that ERAS should be understood not as a set of isolated interventions but as an integrated perioperative care model. Improved outcomes appear to result from coordinated, standardised practices and high levels of adherence, rather than from any single component. Broader neurosurgical experience further indicates a dose-response relationship between pathway adherence and clinical outcomes, underscoring that the quality of implementation is at least as important as the specific elements included. Accordingly, institutions seeking to maximise the benefits of ERAS in spine surgery should emphasise multidisciplinary collaboration, structured education, and continuous audit and feedback alongside the adoption of evidence-based clinical practices. While LOS reductions are frequently reported, improvements in mobilisation, functional recovery, and patient experience may provide a more meaningful assessment of pathway effectiveness.

In contrast, ERAS in craniotomy is constrained by safety considerations. Neurological monitoring, intracranial dynamics, haemodynamic precision, seizure prevention, and haemorrhagic risk constrain the applicability of traditional ERAS elements.^13, 14, 15, 16,18, 19, 20, 21, 22^ LOS reductions observed in cranial ERAS should therefore be interpreted as markers of improved care coordination rather than direct surrogates of neurological recovery.^15, 16, 17, 18,22^ Greater attention to neurological outcomes, quality of recovery, and patient-reported measures is needed to determine the true clinical impact of cranial ERAS pathways.

### ERAS in Special Neurosurgical Populations

Paediatric, geriatric, pituitary, and vascular neurosurgery further illustrate the need for adaptive ERAS implementation. In paediatric populations, caregiver engagement, thermal stability, blood-loss prevention take precedence over early discharge.^8, 24, 25^ In geriatric patients, frailty assessment, delirium prevention, and individualised metabolic and anticoagulation strategies are central determinants of recovery.^11, 26, 27^ Pituitary surgery ERAS prioritises endocrine and fluid balance,^28^ while aneurysm surgery mandates meticulous haemodynamic and neurological surveillance.^12^

Across these settings, meaningful ERAS outcomes extend beyond LOS to include physiological stability, preserved function, neurological outcomes, avoidance of complications, quality of recovery, and patient or caregiver satisfaction.^12, 13, 14, 15, 16, 17, 18, 19, 20, 21, 22, 23, 24, 25, 26, 27, 28, 29, 30, 31, 32, 33^

### Interpreting Apparent Contradictions

Several findings in the current literature appear contradictory but can be explained by differences in outcome selection and protocol implementation. Whilst LOS is consistently reduced, readmission rates and pain scores are often unchanged.^34, 40^ This suggests that ERAS primarily optimises in-hospital recovery processes rather than post-discharge trajectories. Although multimodal analgesia reliably reduces opioid consumption, improvements in pain scores are variable.^38, 50, 52^ The literature on TXA highlights a tension between strong evidence of efficacy in reducing blood loss and ongoing debate about thromboembolic safety in high-risk populations.^43, 45, 46, 47, 48, 49^ These observations highlight the importance of evaluating ERAS through a broader range of patient-centred outcomes rather than relying predominantly on LOS-based measures.

### Implications for Practice and Research

**Figure 2:**
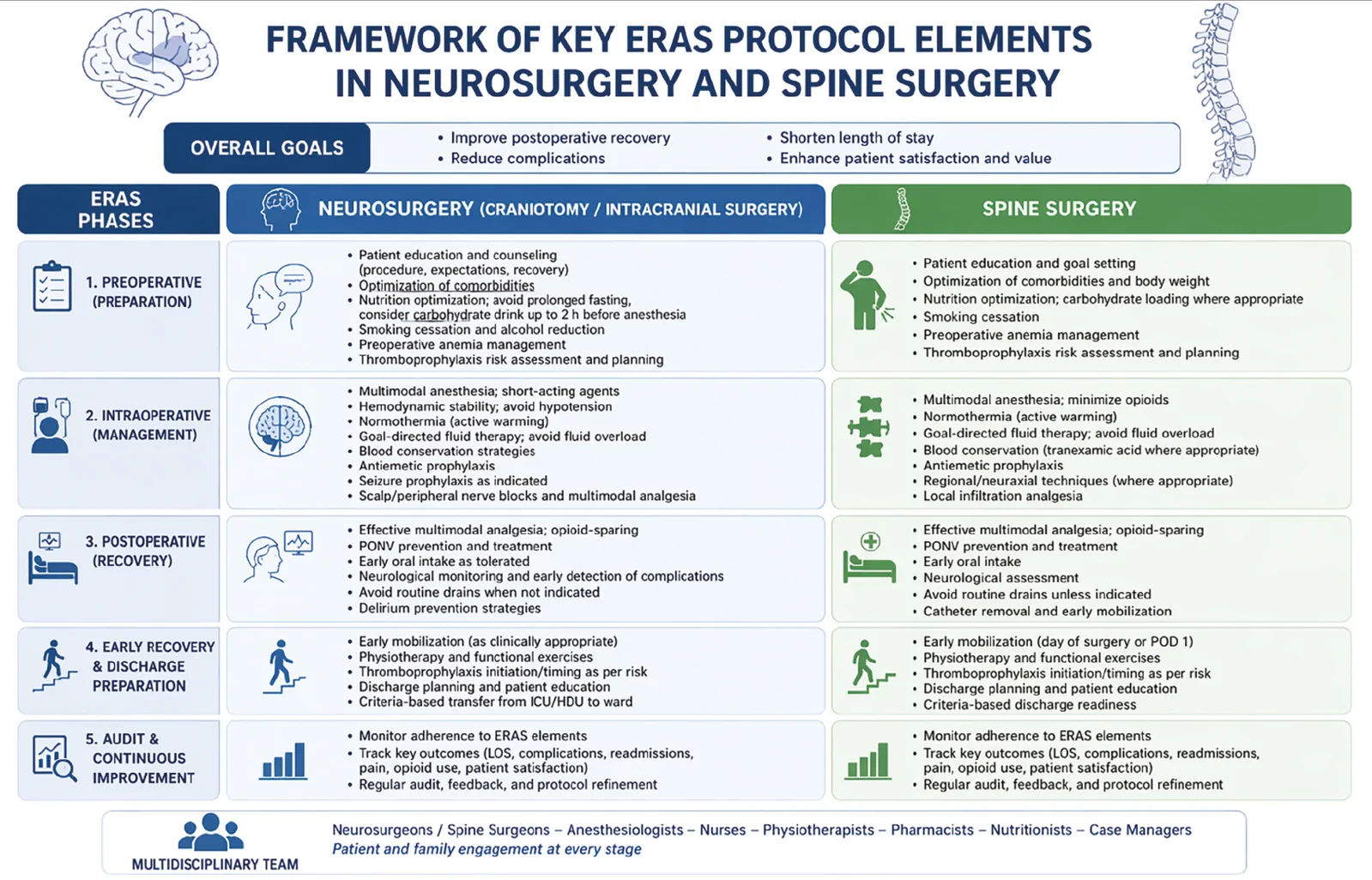
The framework summarizing all specific ERAS protocol elements in neurosurgery and spine surgery. ERAS, enhanced recovery after surgery; ICU, intensive care unit; LOS, length of stay.

**Figure 3:**
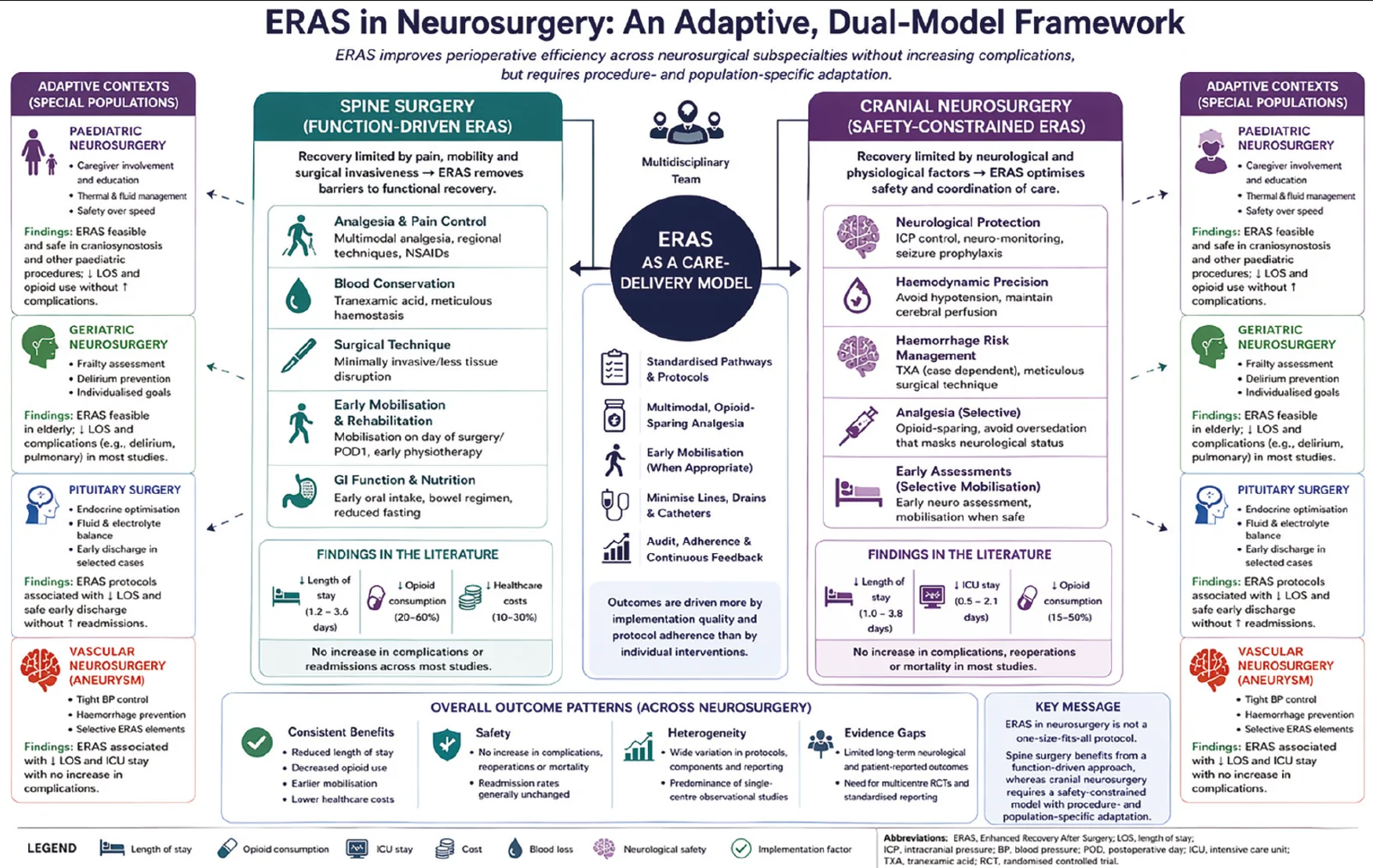
Adaptive ERAS framework in neurosurgery: function-driven and safety-constrained pathways. ERAS, enhanced recovery after surgery; ICU, intensive care unit; LOS, length of stay.

From a clinical perspective, these findings support broader ERAS adoption in neurosurgery, provided that implementation is deliberate, multidisciplinary, and adaptable.^36, 40^ Future research should prioritise multicentre trials, standardised reporting of ERAS components and adherence to them, and greater emphasis on patient-reported outcomes, quality of recovery, functional status, and long-term neurological outcomes.^16, 22, 40^ Such measures are likely to provide a more comprehensive assessment of the true value of ERAS pathways than hospital LOS alone. Figures 2 and 3 summarises key ERAS protocol elements for neurosurgery and spine surgery.

### Clinical Implications and Practical Recommendations

Current evidence suggests that ERAS implementation in neurosurgery should focus on a limited number of high-impact interventions rather than uniformly adopting all traditional ERAS elements. Across both cranial and spinal procedures, the components most consistently associated with improved outcomes include preoperative patient education, multimodal opioid-sparing analgesia, prevention of PONV, maintenance of normothermia, goal-directed fluid management, early nutrition, early mobilization, and multidisciplinary coordination. The benefits of ERAS appear to result primarily from the coordinated application of these measures within a standardized pathway rather than from any single intervention.

In spine surgery, ERAS primarily aims to accelerate recovery and restore functional independence. The most influential components include multimodal analgesia, perioperative blood conservation strategies such as TXA administration, avoidance of unnecessary drains and urinary catheters, and structured early mobilization. These measures facilitate earlier ambulation, reduce opioid-related adverse effects, shorten the hospital stay, and promote a faster return to daily activities.

In cranial neurosurgery, ERAS pathways must balance recovery enhancement with neurological safety. Key interventions include meticulous haemodynamic management, structured neurological monitoring, opioid-sparing analgesia, prevention of PONV, and mobilization after neurological stability has been established. Accordingly, success should be evaluated not only by LOS but also by preservation of neurological function, functional independence, cognitive recovery, and quality of life.

Special populations require procedure-specific adaptations. In paediatric neurosurgery, caregiver involvement, temperature management, and blood conservation are particularly important. In geriatric patients, frailty assessment, nutritional optimization, delirium prevention, and early mobilization are likely to have the greatest impact on recovery. Pituitary surgery requires careful endocrine and fluid-electrolyte management, whereas aneurysm surgery depends heavily on strict haemodynamic control and neurological surveillance.

For institutions implementing neurosurgical ERAS programs, priority should be given to patient education, multimodal analgesia, maintenance of physiological homeostasis, early mobilization, minimization of unnecessary invasive devices, and continuous multidisciplinary audit. Successful implementation requires close collaboration among surgeons, anaesthesiologists, nurses, physiotherapists, nutrition specialists, and rehabilitation teams. Institutions should adopt standardized perioperative pathways, monitor protocol adherence through regular audit and feedback, and adapt protocols according to procedure-specific requirements and local resources.

Importantly, outcome assessment should extend beyond hospital LOS and include measures of functional recovery, neurological outcomes, patient-reported outcomes, complication rates, and quality of life. These metrics more accurately reflect the true clinical value of ERAS and its potential to improve long-term recovery and patient-centred outcomes in neurosurgical practice.

## Conclusion

Current evidence supports ERAS as a safe and effective perioperative care framework for selected neurosurgical populations when implemented with procedure-specific adaptations. Across neurosurgical subspecialties, these pathways enhance recovery efficiency and reduce opioid exposure without compromising neurological safety.

However, ERAS should not be applied as a uniform protocol across all neurosurgical procedures. Rather, it should be understood as a flexible care model that upholds core recovery principles while adapting to procedure-specific physiological demands and safety considerations, particularly for cranial surgery and vulnerable patients.

Future progress will depend on robust, multicentre evaluation; standardised reporting of ERAS components and of adherence; and a stronger focus on long-term neurological and patient-reported outcomes. Ultimately, the success of ERAS in neurosurgery will rely on thoughtful, adaptive implementation in which the quality and consistency in application take precedence over rigid standardisation across diverse clinical settings.

## Figures and Tables

**Table 1. Summarized Level of Recommendation and Evidence for All ERAS Protocol Elements13:** 

**Intervention/ERAS item**	**Evidence level**	**GRADE recommendation**
Preoperative counselling	Low	Strong
Preoperative smoking & alcohol cessation	Moderate	Strong
Preoperative enteral nutrition/immunonutrition	Moderate	Strong/weak
Preoperative fasting and carbohydrate loading	Low	Strong
Antithrombotic prophylaxis	High	Strong
Scalp shaving	Moderate	Weak against
Antibiotic prophylaxis	High	Strong
Scalp infiltrations & blocks	Moderate	Strong
Anaesthetic protocol choices	Variable	Mixed/conditional
Non‑opioid analgesia	Mixed	Mixed
PONV prophylaxis	Various	Strong for certain agents
Minimally invasive craniotomies	Very low	Weak
Normothermia/hypothermia avoidance	High	Strong
Fluid balance monitoring	Low	Strong
Urinary drainage	Moderate	Strong
Postoperative artificial nutrition	Moderate	Strong
Early mobilization	High	Strong
Audit	Moderate	Strong

ERAS, enhances recovery after surgery; PONV, postoperative nausea and vomiting

**Table 2. Individual Studies on ERAS Protocol for Craniotomy:** 

**Study**	**Country**	**Design**	**n**	**Comparator**	**Key ERAS components (structured)**	**Key findings**	**Major limitations**
Hagan et al.,^13^	USA	Narrative proposal	N/A	None	Preop counselling; carb loading; multimodal analgesia; thromboprophylaxis; normothermia; early mobilization	Proposed 17-component neurosurgical ERAS framework	Conceptual; no clinical data
Liu et al.,^19^	China	RCT	140 (70/70)	Conventional care	Preop education; carb loading; absorbable sutures; multimodal analgesia; PONV prophylaxis; early mobilization	Improved patient satisfaction; shorter LOS; no ↑ complications	Single centre; unblinded
Qu et al.,^20^	China	RCT	129 (64/65)	Conventional care	Multimodal analgesia; scalp block; PONV prophylaxis; early mobilization; early feeding	Reduced pain scores; shorter LOS	Single centre; discharge bias possible
Elayat et al.,^14^	India	Non-randomized controlled trial	70	Conventional care	Family education; carb loading; scalp block; limited opioids; GDFT; early catheter removal; thromboprophylaxis	Reduced ICU stay; no significant LOS difference	Non-randomized; confounding risk
Feng et al.,^15^	China	Retrospective cohort	100 (50/50)	Conventional care	Education; carb loading; scalp block; PONV prevention; early mobilization; early catheter removal	Improved recovery indicators; no ↑ complications	Retrospective; moderate sample
Wang et al.,^17^	China	Controlled implementation study	151 (80/71)	Time-separated conventional cohort	Preop optimization; carb loading; GDFT; scalp block; early drain removal; early feeding; thromboprophylaxis	Reduced LOS; earlier mobilization & oral intake; no ↑ complications	Not fully randomized; glioma-specific
Wang et al.,^18^	China	RCT	151 (76/75)	Conventional care	Education; carb loading; GDFT; multimodal analgesia; PONV prophylaxis; early mobilization; thromboprophylaxis	Reduced LOS, pain, PONV, and hospitalization cost	Single centre; no long-term outcomes
Chen et al.,^21^	China	Prospective single-arm feasibility	20	None	Monitored anaesthesia care; scalp nerve block; opioid minimization; early recovery focus	Demonstrated feasibility of ERANS in awake craniotomy	No comparator; small sample
Kaewborisutsakul et al.,^16^	Thailand	Retrospective adherence study	100	High vs low adherence	Education; carb loading; multimodal analgesia; minimal shaving; early feeding; thromboprophylaxis	Higher adherence associated with improved LOS and recovery	Observational; confounding risk
Zangi et al.,^22^	Iran	Systematic review	10 studies	N/A	Synthesized multimodal ERAS elements across trials	Reported reduced LOS; highlighted protocol heterogeneity	Predominantly single-centre data; heterogeneous designs

LOS, length of stay; ERANS, enhanced recovery after neurosurgery; PONV, postoperative nausea and vomiting; ERAS, enhanced recovery after surgery; GDFT, goal-directed fluid therapy; RCT, randomized controlled trial; ICU, intensive care unit; USA, United States of America; N/A, not applicable

**Table 3. Specific ERAS Element for Spine Surgical Procedures:** 

**Preoperative**	**Intraoperative**	**Postoperative**
Education	Minimally invasive spine surgery	Early mobilization
Smoking cessation	Multimodal analgesia	Early oral intake
Anaemia correction	Minimize bleeding (blood pressure control, TXA)	-
Preoperative chronic medication and diseases optimization	Hypothermia, PONV, infection prophylaxis	-
Nutritional supplementation	Minimize use of drains, urinary catheter	-

ERAS, enhanced recovery after surgery; PONV, postoperative nausea and vomiting; TXA, tranexamic acid
